# Chamäleon Lemierre-Syndrom?

**DOI:** 10.1007/s00101-026-01689-9

**Published:** 2026-05-07

**Authors:** Oliver Sommerfeld, Sebastian Reinartz, Michael Bauer, Petra Dickmann

**Affiliations:** 1 Klinik für Anästhesiologie und Intensivmedizin, Universitätsklinikum Jena, Am Klinikum 1, 07747 Jena, Deutschland; 2 Institut für Diagnostische und Interventionelle Radiologie, Universitätsklinikum Jena, Jena, Deutschland; 3 Klinik für Notfallmedizin, Universitätsklinikum Jena, Jena, Deutschland

## Anamnese

Die Vorstellung eines 25-jährigen männlichen Patienten in unserer zentralen Notaufnahme (ZNA) erfolgte durch Hausarzteinweisung wegen progredienter AZ(Allgemeinzustands-)-Verschlechterung, Belastungsdyspnoe und gelblich verfärbtem Hautkolorit (Ikterus). Anamnestisch bestanden seit einer Woche ein Atemwegsinfekt mit grippalen Symptomen, linksbetonte Halsschmerzen, Husten und Fieber bis 39 °C. Eigenanamnestisch waren ein Asthma bronchiale seit Kindheit, mäßiger Alkohol- und Nikotinkonsum bekannt. Die Medikamentenanamnese war leer, in der Reiseanamnese fand sich zuletzt ein Urlaub in Kroatien.

## Klinischer Untersuchungsbefund und Diagnostik in der ZNA

In der ZNA zeigte sich klinisch das Bild einer schweren Sepsis mit Tachypnoe (Atemfrequenz 40/min), Tachykardie (Herzfrequenz 115/min), febriler Temperatur (38,3 °C) und beginnend hypotensiven Kreislaufparametern (RR 90/57 mm Hg). In der körperlichen Untersuchung fanden sich linksseitig Zeichen einer Tonsillitis mit schmerzhaften Lymphknoten der Regio III/IV, leicht gerötete Tonsillen ohne Kieferklemme sowie ein ausgeprägter Ikterus. Der übrige Status war unauffällig. Laborchemisch imponierten sehr hohe Entzündungsmarker (PCT 133,6 ng/ml, CRP 138 mg/l, Leukozyten 14,1 Gpt/l) sowie Retentionsparameter eines akuten Nierenversagens (Kreatinin 240 µmol/l). Zusätzlich zeigten sich deutlich erhöhte Transaminasen (ASAT 3,13 µmol/l·s, ALAT 2,28 µmol/l·s) und Bilirubinwerte (Gesamtbilirubin 187 µmol/l) sowie eine ausgeprägte Thrombopenie (Thrombozyten 15 Gpt/l). Der Verlauf der Laborparameter ist in Tab. 1 dargestellt.

**Tab. 1:** Laborparameter im klinischen Verlauf Aufnahme, Tag 1 bis 3 und letzter Wert.

Laborparameter	Aufnahme	Tag 1	Tag 2	Tag 3	Letzter Wert	Referenz
*CRP (mg/l)*	138,0	138,1	56,4	38,1	29,6	*<* *5,0*
*PCT (ng/ml)*	133,6	45,2	20,3	7,02	1,49	*<* *0,5*
*Leukozyten (Gpt/l)*	14,1	15,2	13,5	11,3	5,8	*4,4–11,3*
*Thrombozyten (Gpt/l)*	15	15	45	83	526	*150–360*
*Kreatinin (µmol/l)*	240	137	92	80	61	*62–106*
*Bilirubin (µmol/l)*	187	177	67	48	20	*<* *21,0*
ASAT (µmol/l*s)	3,13	1,6	0,58	0,47	0,32	*<* *0,85*
ALAT (µmol/l*s)	2,28	1,77	1,08	0,88	0,77	*<* *0,83*
AP (µmol/l*s)	4,54	2,63	1,95	–	1,89	*0,67–2,15*
GLDH (µmol/l*s)	0,329	0,288	0,089	0,047	0,064	*<* *0,12*
*Laktat, (venös mmol/l)*	1,7	1,3	0,8	1,4	1,4	*0,5–2,2*
*SOFA*	11	10	6	–	6	–

*CRP* C-reaktives Protein, *PCT* Procalcitonin, *ASAT* Aspartat-Aminotransferase, *ALAT* Alanin-Aminotransferase, *AP* Alkalische Phosphatase, *GLDH* Glutamatdehydrogenase, *SOFA* Sequential Organ Failure Assessment

**Abb. 1:**
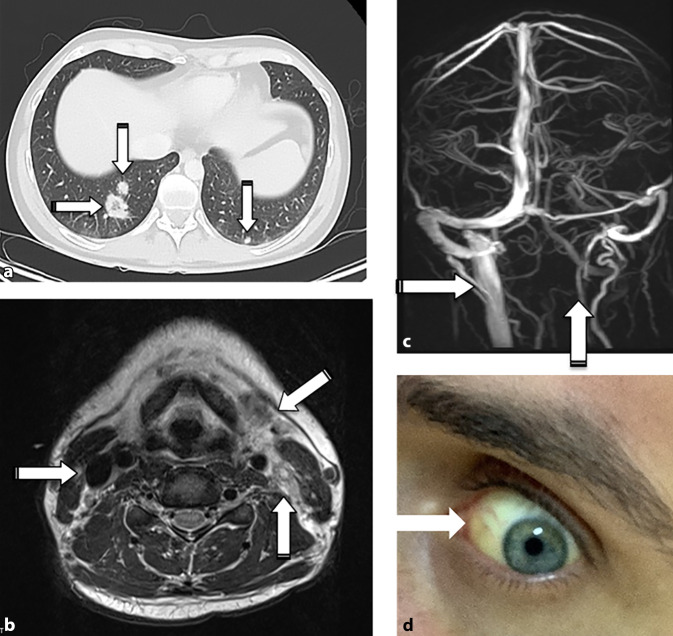
**a** CT der Lunge mit bilateralem Nachweis multipler, a.e. septischer Embolien (*Pfeile*). **b** Axiale, T2-gewichtete Turbo-Spin-Echo-Sequenz des Halses: Thrombose der V. jugularis sinistra wegen fehlendem Flow-Void (*vertikaler Pfeil*), regelrecht durchflossene V. jugularis dextra (*horizontaler Pfeil*). Lymphadenopathie (*schräger Pfeil*). **c** Koronare Maximumintensitätsprojektion der 3D-Phasenfllussmessung der zerebralen Sinus und Venen mit ausbleibendem Signal in der okkludierten V. jugularis sinistra (*vertikaler Pfeil*) und regelrechter Kontrastierung kontralateral (*horizontaler Pfeil*) sowie der Sinus. **d** Klinisches Bild bei Untersuchung auf Intensivstation mit Sklerenikterus (*Pfeil*)

Zur Fokussuche wurden mikrobiologische Proben (Blut, Urin und Sputum) gewonnen. Zusätzlich erfolgte die erweiterte Diagnostik auf Tuberkulose, Hantaviren, Leptospiren, CMV, EBV, Hepatitiden (B, C und E) sowie Autoimmundiagnostik. Innerhalb von 20 min wurde nach initialer Basisdiagnostik bei schwerer Sepsis mit beginnendem Schock in der ZNA eine Kreislaufstabilisierung mit Noradrenalin sowie eine Volumen- und Antibiotikatherapie (Piperacillin/Tazobactam und Clindamycin) begonnen. Zudem wurde bei Sepsis ohne klaren Fokus von hier aus zügig eine kontrastmittelverstärkte CT von Hals, Thorax, Abdomen und Becken durchgeführt. Es zeigten sich multiple pulmonale Konsolidierungen mit Einschmelzungen (Abb. 1a) im Sinne septischer Embolien, eine linksseitige Lymphadenopathie ohne Abszedierung (Abb. 1b), ein langstreckiger Verschluss der Vena jugularis interna (VJI) links (Abb. 1b, c) sowie eine Hepatosplenomegalie. Die Duplexsonographie der Halsgefäße bestätigte den Verdacht auf Thrombose der linken VJI. Die orientierende Echokardiographie ergab keinen Hinweis auf eine Endokarditis. Bei nachgewiesener Venenthrombose erfolgte ergänzend eine MRT von Kopf und Hals ohne Nachweis eines intrakraniellen Fokus oder einer Sinusvenenthrombose.

## Intensivtherapie und Verlauf

Aufgrund der laborchemischen Hinweise auf Leberdysfunktion mit Ikterus (Abb. 1d), Thrombopenie und akutem Nierenversagen wurde der Patient zur weiteren Überwachung innerhalb von 2 h von der ZNA für die erweiterte Therapie auf die Intensivstation aufgenommen. Konsiliarisch wurden die Kolleg:innen der Infektiologie (schwere Infektion) und HNO-Heilkunde (Verdacht auf Peritonsillitis) hinzugezogen. Die Kreislauf- und Volumentherapie wurde auf der Intensivstation fortgeführt. Vorübergehend erfolgte eine Therapieeskalation mit Vasopressin und Steroidgabe (Hydrokortison/Fludrokortison). Zusätzlich wurden insgesamt 30 g Immunglobuline (Pentaglobulin) über zwei Tage bei schwerem septischen Schock appliziert. Unter dieser Therapie kam es rasch zur Stabilisierung der hämodynamischen Situation. Am Folgetag wurde in den Blutkulturen *Fusobacterium necrophorum* nachgewiesen. In Zusammenschau der Befunde – Peritonsillitis ohne Abszess, septische pulmonale Embolien, langstreckige Thrombose der linken VJI ohne intrakranielle Ausdehnung sowie Nieren- und Leberversagen mit Ikterus und Thrombopenie – wurde die Diagnose eines Lemierre-Syndroms gestellt [1–3, 6]. Weitere mikrobiologische Untersuchungen ergaben keinen alternativen Fokus. Bei nachgewiesener Thrombose wurde eine therapeutische Antikoagulation zunächst mit unfraktioniertem Heparin begonnen, nach Besserung der Nierenfunktion mit niedermolekularem Heparin (Tinzaparin) fortgesetzt und im Verlauf auf ein NOAK (Apixaban) für insgesamt drei Monate umgestellt. Leber- und Nierenfunktion normalisierten sich rasch. Am dritten Tag nach Aufnahme war der Patient hämodynamisch stabil und konnte auf die Normalstation verlegt werden. Im weiteren Verlauf wurde die Antibiotikatherapie zunächst auf Penicillin G deeskaliert. Nach insgesamt zehn Tagen stationärer Behandlung und weitgehender Normalisierung der Laborparameter konnte der Patient in gutem Allgemeinzustand entlassen werden. Poststationär wurde eine orale Antibiose mit Clindamycin über weitere drei Wochen fortgeführt. Zudem wurde eine elektive Tonsillektomie im Intervall von circa sechs Wochen geplant.

## Diskussion

Das Lemierre-Syndrom ist eine seltene, potenziell letale Komplikation einer Infektion des Mund-Rachen-Raums, typischerweise Tonsillopharyngitis, mit septischer Thrombophlebitis der Vena jugularis interna (VJI) und septischen Embolien, überwiegend in die Lunge [1–3, 6]. Klassischerweise umfasst das Syndrom die Trias aus Oropharynxinfektion, Bakteriämie durch *Fusobacterium necrophorum* und jugularvenöser Thrombose mit metastatischen Abszessen [1–3, 6]. Im vorliegenden Fall traten früh ein ausgeprägter Ikterus, Leber- und Nierenversagen sowie Thrombopenie auf, wie sie bei schweren Verläufen beschrieben sind und die hohe systemische Entzündungsaktivität widerspiegeln [1–3, 6]. Die initiale kalkulierte breite Antibiose mit Piperacillin/Tazobactam und Clindamycin erfolgte aufgrund der klinischen Zeichen eines septischen Schocks und des möglichen Keimspektrums im oropharyngealen Bereich. Ohne frühzeitige Diagnose und adäquate Therapie ist die Mortalität historisch hoch; moderne Fallserien berichten jedoch über deutlich verbesserte Prognosen bei rascher Diagnostik, kalkulierter antimikrobieller Therapie und intensivmedizinischer Versorgung. Zusätzlich sind chirurgische Interventionen bei septischen Embolien v. a. beispielweise bei Empyemen oder Mastoiditis sowie in selten Fällen Ligation der VJI als therapeutische Optionen bei fehlender Stabilisierung unter Antibiose in der Literatur beschrieben [1–3, 6].

In diesem Fall war der Ikterus initiales Warnsignal und Anlass für die umgehende Vorstellung in der Klinik. Die strukturierte Sepsisdiagnostik mit frühzeitigem „bundle-orientierten“ Vorgehen – inklusive Laboranalytik mit Sepsis- und Organfunktionsparametern, unmittelbarer Blutkulturabnahme, erweiterter mikrobiologischer Diagnostik sowie kontrastmittelverstärkter Bildgebung von Hals, Thorax, Abdomen und Becken – ermöglichte eine rasche Identifikation der charakteristischen Befundkonstellation. Dieses Vorgehen entspricht den empfohlenen diagnostischen Schritten bei Verdacht auf Lemierre-Syndrom, bei dem die Bildgebung der Halsgefäße (CT, MRT, Duplexsonographie) zur Sicherung der jugularvenösen Thrombose zentral ist [1–3]. Die ergänzende MRT des Kopf-Hals-Bereichs diente dem Ausschluss intrakranieller Komplikationen, die in der Literatur als prognostisch ungünstig beschrieben sind [3, 4].

Die ausgeprägte Thrombopenie, der Ikterus sowie das gleichzeitige Leber- und Nierenversagen entsprechen bekannten, wenn auch nicht obligaten Organmanifestationen des Lemierre-Syndroms und spiegeln die hohe systemische Entzündungsaktivität wider [1–3, 6]. Bereits Lemierre beschrieb neben Pharyngitis, septischer Thrombophlebitis und pulmonalen Embolien schwere systemische Verläufe; neuere Fallserien und Reviews verknüpfen solche Konstellationen mit erhöhter Morbidität und Mortalität, insbesondere bei verzögerter Diagnosestellung und verspätetem Therapiebeginn [1–3, 6]. Im vorliegenden Fall konnten die Organfunktionsstörungen durch eine aufmerksame Identifikation eines kritisch kranken Patienten in der Notaufnahme, eine frühzeitige intensivmedizinische Behandlung und eine zielgerichtete antimikrobielle Therapie rasch rückläufig gestaltet werden [3, 4].

Besonders relevant ist die schnelle und koordinierte Zusammenarbeit mehrerer Disziplinen: Notaufnahme, Intensivmedizin, Radiologie, Infektiologie und HNO-Heilkunde agierten parallel und nicht sequenziell. Dies ermöglichte, hämodynamische Stabilisierung, leitliniengerechte kalkulierte Antibiose mit anaerober Abdeckung, Fokussuche und Planung der elektiven Tonsillektomie frühzeitig zu verzahnen – ein Vorgehen, das in aktuellen Übersichtsarbeiten als entscheidend für eine Prognoseverbesserung beim Lemierre-Syndrom beschrieben wird [1–3].

Die Rolle der Antikoagulation bei jugularvenöser Thrombose im Rahmen des Lemierre-Syndroms ist weiterhin nicht durch randomisierte Studien gesichert. Metaanalysen und systematische Übersichten zeigen keinen eindeutigen Mortalitätsvorteil, berichten jedoch über eine insgesamt gute Sicherheit und potenziell günstigere Verläufe bei ausgedehnter Thrombose, progredienter Embolisation oder intrakranieller Ausdehnung [3, 7–9]. Die in diesem Fall gewählte Strategie mit initialer Antikoagulation mittels niedermolekularem Heparin und anschließender oraler Antikoagulation über drei Monate entspricht einem individualisierten, aber in der Literatur gut abgebildeten Vorgehen [3, 7–10].

Epidemiologische Daten deuten darauf hin, dass viele Betroffene vor Diagnosestellung mehrfach medizinische Kontakte haben, häufig ohne adäquate oder zeitgerechte Antibiotikatherapie. Der Fall unterstreicht daher die Notwendigkeit, bei jungen Patientinnen und Patienten mit Pharyngitis/Tonsillitis, persistierendem oder progredientem Krankheitsgefühl, respiratorischer Symptomatik und Zeichen einer Sepsis frühzeitig an ein Lemierre-Syndrom zu denken. Aus anästhesiologisch-intensivmedizinischer Perspektive ist die frühzeitige Identifikation solcher „Chamäleon-Verläufe“ und ein strukturiertes interdisziplinäres Management entscheidend, um eine rasche Stabilisierung zu erreichen und langfristige Organschäden zu vermeiden [1–3, 5, 6].

## Fazit für die Praxis

- Das Lemierre-Syndrom ist eine seltene, potenziell letale Komplikation nach Pharyngitis/Tonsillitis bei jungen Patient:innen.
- Warnsignale sind Fieber, Halsschmerzen, respiratorische Symptome und Sepsiszeichen mit Organversagen.
- Entscheidend sind frühe Diagnosestellung, interdisziplinäres Management und rascher Beginn einer kalkulierten, anaerob wirksamen Antibiotikatherapie.

## Data Availability

Beim vorliegenden Artikel handelt es sich um einen Fallbericht. Die Zustimmung zur Publikation durch den Patienten ist vorliegend. Es erfolgt keine Weitergabe der Daten an Dritte.
